# An analysis of national collaboration with Spanish researchers abroad in the health sciences

**DOI:** 10.1186/s12961-015-0055-2

**Published:** 2015-11-07

**Authors:** Pedro Aceituno-Aceituno, Sonia Janeth Romero-Martínez, Patricia Victor-Ponce, José García-Núñez

**Affiliations:** Departamento de Administración y Dirección de Empresas y Economía, Universidad a Distancia de Madrid (UDIMA), Carretera de La Coruña, km 38,500 – Vía de servicio, n.° 15, 28400 Collado Villalba, Madrid Spain; Departamento de Psicología, Universidad a Distancia de Madrid (UDIMA), Collado Villalba, Madrid Spain

**Keywords:** Brain gain, Collaboration, Education, Knowledge, Return, Research career

## Abstract

**Background:**

The establishment of scientific collaborations with researchers abroad can be considered a good practice to make appropriate use of their knowledge and to increase the possibilities of them returning to their country. This paper analyses the collaboration between Spanish researchers abroad devoted to health sciences and national science institutions.

**Methods:**

We used the Fontes’ approach to perform a study on this collaboration with Spanish researchers abroad. We measured the level of national and international cooperation, the opportunity provided by the host country to collaborate, the promotion of collaboration by national science institutions, and the types of collaboration. A total of 88 biomedical researchers out of the 268 Spanish scientists who filled up the survey participated in the study. Different data analyses were performed to study the variables selected to measure the scientific collaboration and profile of Spanish researchers abroad.

**Results:**

There is a high level of cooperation between Spanish health science researchers abroad and international institutions, which contrasts with the small-scale collaboration with national institutions. Host countries facilitate this collaboration with national and international scientific institutions to a larger extent than the level of collaboration promotion carried out by Spanish institutions.

**Conclusions:**

The national collaboration with Spanish researchers abroad in the health sciences is limited. Thus, the practice of making appropriate use of the potential of their expertise should be promoted and the opportunities for Spanish health science researchers to return home should be improved.

**Electronic supplementary material:**

The online version of this article (doi:10.1186/s12961-015-0055-2) contains supplementary material, which is available to authorized users.

## Background

In 1963, the Royal Society [1] defined brain drain as the migration of British scientists to the United States, with the term being subsequently used more widely to describe the migration of professionals and scholars from developing to developed countries, seriously compromising the economy of developing countries and providing unfair technological advantages to developed countries [2]. At the end of the 80s and beginning of the 90s, several studies regarding the mobility of scientists radically modified the perspective of the loss of a highly skilled labour force. On the contrary, the assumption that scientists and technologists produce knowledge at a global level network facilitated the adoption of a brain-gain approach to put local scientists in contact with the expatriates [3]. The brain gain approach can also be favoured by the intensive use of information and communication technologies to develop virtual networks of investigation that complement onsite networks [4]. Some countries have established policies of this type for their qualified personnel, and especially for their scientists, e.g. in Argentina (Red de Argentinos Investigadores y Científicos en el Exterior –RAICES) [5] or Colombia (Red Caldas de Investigadores en el Exterior) [6].

One of the advantages of the brain-gain approach is the easiness for national scientists to continue yielding benefits to their countries of origin without losing their professional positions in their destination countries, while also being economically affordable for their countries of origin [7]. In this regard, large investments are not necessary, but rather existing resources must be capitalised. As a result, any country wanting to make these social, political, organisational and technical efforts can access not only the knowledge of their expatriates, but also their professional networks abroad [8, 9].

A long-term stay of researchers overseas (3 years or more) has a negative impact on the collaboration networks with the country of origin [10]. This is because the number of relationships created abroad exceeds the ones kept with the national network, and with this the drain goes from being transitory to permanent [11]. Indeed, the probabilities of returning as well as the scientific productivity in the host country are greater when researchers keep contacts in their country of origin [12]. Maintaining a close collaboration with the country of origin while being abroad is a basic practice used to stop the researcher from fleeing to their host country forever [13]. As a result, scientists who do return, often go back to the same organisation [14]. In fact, Fontes demonstrates that every researcher who returns kept ties with their institution of origin [15]. However, establishing these connections might not be easy. It is, therefore, interesting to further study these aspects relative to collaboration to report back to the political authorities and others agents interested in improving the benefits of scientific mobility.

The concept of collaboration, as used herein, is defined as the achievement of joint actions between national scientific institutions and Spanish researchers abroad to make good use of their knowledge and to increase their possibilities of return.

To foster these partnerships, several scientific societies abroad have recently been created in Spain with the support of the Fundación Española para la Ciencia y la Tecnología (FECYT) including the Society of Spanish Researchers in the United Kingdom/Comunidad de Científicos Españoles en el Reino Unido (SRUK/CERU) [16], the Comunidad de Científicos Españoles en la República Federal de Alemania (CERFA) [17], the Asociación de Científicos Españoles en Suecia (ACES) [18] and the Comunidad de Españoles Científicos en Estados Unidos (ECUSA) [19]*.* These associations’ additional important functions are to create a social network for national researchers in the host country, increase social awareness of the value of science, act as the scientists’ voice, and foster collaboration between national and international scientific institutions with Spanish scientists abroad.

There are no official data of the figure of Spanish researchers abroad. The last census was conducted in 2010 by the former Sistema de Comunicación con Investigadores en el Exterior (RedIEX) of FECYT, with around 1,300 Spanish researchers registered. Most of them involved in research in health sciences (44%), followed by physics and engineering (28%), life sciences (13%), social sciences (7%) and humanities (8%). The fundamental reasons for departure were greater prospects of having a research career and salaries [20]. In particular, for health science researchers, additional reasons can be the lack of structure and recognition of a research career [21–27], their high level of education [21, 23], the importance of improving the management of staff so that doctors wanting to conduct research have the time and emotional space to do so [28], and the relevance of establishing science networks in this sector [22, 24, 26]. Regarding the last aspect, strengthening international collaboration with national researchers working in Spain may be important to enrich these national scientists’ investigations without leaving their professional positions in Spain. One example is the collaboration of practitioners in Madrid and New York improving inclusion of underrepresented populations in research (for example, physicians, social workers, or health educators) [29]. Accordingly, different government policy plans have been put in place over the last decade to foster human resources in research and development (R&D), and to promote entrance to labour market, the internationalisation of R&D, and innovation and knowledge transfer [30–33]. Further, in the context of this study, these same plans provide public financial support in national and international mobility and networking for all agents and areas of knowledge in the Sistema Español de Ciencia, Tecnología e Innovación (SECTI). It can therefore be assumed that the scope for improving these aspects is still wide, particularly in health sciences. Therefore, comparing areas of knowledge seems relevant in order to determine possible differences in good practices for specific areas.

That being said, the goal of the present study is to analyse the collaboration between the Spanish researchers abroad dedicated to health sciences and the Spanish scientific institutions as good practice in order to make use of their knowledge and to increase the possibilities of them returning to their home country.

## Methods

A study was performed among Spanish scientists based abroad through an online questionnaire between January and March 2014. This study belonged to an authorized and joint project between the Dirección General de Migraciones del Ministerio de Empleo y Seguridad Social de España and the Universidad a Distancia de Madrid.

### Participants and scope

As discussed above, public financial support of national plans has been uniform within all areas of knowledge in mobility and networking. Therefore, it may be relevant to compare different areas of knowledge to determine possible differences in good practices developed for these specific areas.

Likewise, according to the concept of collaboration used in this study, the participants’ data in this study have been collected by associations of Spanish researchers abroad (SRUK/CERU, CERFA, ACES and ECUSA) since these associations are interested in scientific collaboration with Spain, as discussed above. Each association has its own checklist consisting of members and other Spanish researchers worldwide related to the association. The associations’ total population comprises 2,060 Spanish researchers abroad [34], of which 268 scientists (13%) responded our questionnaire.

From January 13th to March 15th, 2014, these associations contributed to circulating the questionnaire by email to Spanish researchers abroad. Approximately every 14 days, study researchers consulted the associations with regards to the number of answers obtained. With this information, these associations kept on sending this questionnaire by email. Additionally, the associations used their Facebook and social networks (SRUK/CERU, CERFA and ECUSA) to obtain a higher response rate [35]. At the beginning of March, the latest email and calls to social networks were sent. Since then, responses scarcely increased. The questionnaire closed on March 15th, 2014. All participants provided informed consent, as embedded in the questionnaire, to participate in the study and the authors of this study did not interact with the participants in any way. Completion of the questionnaire was voluntary and anonymous.

### Questionnaire

To collect data, a questionnaire was designed following Fontes’ approach [15]. Fontes analysed the return of 55 biotechnology researchers to Portugal. Similar to Spain, Portugal has highly-skilled scientific personnel, but lacks both a very strong national R&D system and consistent data about scientific mobility flow. On the basis of the above, four variables were studied. These items were evaluated using a 7-point Likert scale, where higher values signify a greater degree of collaboration and lower values signify a lower degree.

#### Level of national and international scientific collaboration

This variable is composed of three items to estimate the level of national and international scientific collaboration: (1) the extent of collaboration with some international institution, (2) the extent of collaboration with some Spanish scientific institution, (3) the extent of collaboration with the Spanish scientific institution of origin.

#### Efforts made by the host country to achieve collaboration

Again, this variable is composed of three items to estimate its degree of fostering collaborations with (1) other international institutions, (2) Spanish scientific institutions, (3) the Spanish scientific institution of origin.

#### Efforts made by national scientific institutions to promote collaboration

This variable is composed of only one item to assess the degree of promotion of collaboration by Spanish scientific institutions.

#### Types of collaboration maintained with scientific institutions in Spain

To reflect the nature of the scientific collaboration, this variable was measured by 15 items in a dichotomous format (yes or not). Given the definition of collaboration adopted in this paper, there are four types of essential collaborations to make good use of the knowledge of Spanish researchers abroad and to increase the possibilities of their returning to public scientific institutions: (1) elaborating joint publications, (2) applying for patents, (3) implementing joint research projects, and (4) attending conferences. Further, collaborations such as (5) obtaining research contracts and (6) execution/tutoring of PhD theses can help in this regard. Additionally, since, in order to access vacancies in Spanish public institutions professional experience is increasingly valued, the following aspects can be highlighted: (7) consultancy jobs, (8) informal contacts/business placements, (9) participation in networks by electronic means, (10) spin-off creation, (11) employee training, (12) funding procurement for the Spanish institutions, (13) creation of new or improved products or processes, (14) influence in socio-political changes, and (15) recruiting of researchers for scientific Spanish institutions. All these aspects may also be valued by Spanish companies or other private organizations in order to start internationalization processes and obtain enhanced knowledge of professional activities developed in other countries.

Following the approach by Baruffaldi and Landoni [12], different variables were considered to create the participants profile: areas of knowledge, sex, age, duration of the stay abroad, career stage, host country, the reasons to move out from the national country and intention and possibility of returning to continue with scientific career in Spain. Two more variables were added to take into account personal reasons: civil status and paternity/maternity.

### Questionnaire properties and development

In order to ensure quality, this survey was developed according to the following steps: (1) choice of the method to approach respondents (in this case, email and social networks), (2) selection and definition of the variables to measure, (3) identification of the items in each variable, (4) description of the instructions, and (5) deployment of a pilot test of the survey draft.

The pilot test focused on the questionnaire’s psychometric properties. In order to check the validity of the obtained scores, the questionnaire was given to a group of ten researchers in different areas. After being informed of the objectives and variables definition, they were asked to respond it. Based on their answers, the following aspects were addressed: (1) were the questions clear enough? (2) is there any ambiguity in the questions? (3) is there any important issue missing? (4) do questions correspond to the objectives of each variable? (5) are there additional questions to improve the survey and/or its results?

This checking motivated us to include some questions not originally considered in our draft, for example those concerning their reasons to move and their intention to return. All the researchers in the checking exercise considered that all the questions were clear and without any ambiguity.

Considering the character of the variables ‘Profile of Spanish researchers abroad’ and ‘Efforts made by national scientific institutions to promote collaboration’, it is unnecessary to address their reliability. The first one is composed of independent sociodemographic variables whereas the second comprises only one item. To test reliability for the variables ‘Level of national and international scientific collaboration’ and ‘Efforts made by the host country to achieve collaboration’, the two-half method was used due to the small number of items these variables consisted of. The results for the first variable indicate a high correlation between test forms (0.64); the Spearman-Brown coefficient was 0.80 and the two halves of Guttman 0.74, indicating an adequate reliability. The second of these variables presents better reliability, with a high correlation between test forms of 0.78, a Spearman-Brown coefficient of 0.89 and the two halves of Guttman 0.86. Finally, the reliability of the variable ‘Types of collaboration maintained with scientific institutions in Spain’ was estimated by internal consistency (Cronbach Alpha), which shows to have a high value (α = 0.78).

### Study design and data analysis

The study was both quantitative and cross-sectional, with an associative and comparative strategy design [36]. Two types of data analysis were carried out. For the variables ‘Level of national and international scientific collaboration’, ‘Efforts made by the host country to achieve collaboration’ and ‘Efforts made by national scientific institutions to promote collaboration’, an inferential analysis was carried out with the aim of comparing the average ranks of formed groups according to four different areas of knowledge: social and legal sciences and humanities, sciences, health sciences, and engineering and architecture. This inferential analysis was made with a non-parametric statistic (Kruskal–Wallis test) due to the failure of the normality assumption or because of the ordinal nature of the data. In the case of the variables ‘Types of collaboration maintained with scientific institutions in Spain’ and ‘Profile of Spanish researchers abroad’ a descriptive analysis was used, including graphics, frequencies and percentage analyses. The analyses were conducted using SSPS 17.0 software.

## Results

### Level of national and international scientific collaboration

As discussed above, a Kruskal–Wallis test was conducted in order to compare the average ranks of the four groups in seven questions relative to the three first variables. The Kruskal–Wallis test showed that the only significant difference was observed in the collaboration degree with the Spanish institution of origin (*χ*^2^ = 10.606, *P* <0.05; Table 1). To gain further insight into this result, the post-hoc contrast was applied with the Mann–Whitney test. These analyses indicate that the main differences lay between health sciences versus social and legal sciences and humanities (*U* = 340, *Z* = −2.575, *P* = 0.010, *r* = 0.123) and between social and legal sciences and humanities versus engineering and architecture (*U* = 121, *Z* = −2.647, *P* = 0.008, *r* = 0.123); there were no significant differences between other pairs of areas. These results indicate that researchers in the health sciences have a lower degree of collaboration with their Spanish scientific institution of origin than their colleagues in the social and legal sciences and humanities (average rank of 48.36 and 68.85, respectively). Similarly, researchers in engineering and architecture had a lower degree of collaboration compared with social and legal sciences and humanities researchers (average rank of 21.06 and 31.69, respectively). In view of these results, Kruskal–Wallis and Mann–Whitney post-hoc tests showed that there were significant statistical differences between researchers in social and legal sciences and humanities and those in other areas of knowledge (health sciences and engineering and architecture), but only in the degree of collaboration with the Spanish institution of origin.

**Table 1:** **National and international collaboration**

**a)** ***Level of national and international scientific collaboration***					
**1. To which extent are you collaborating in your research activity with some international institution?**	**Median**	**Average rank**	***χ*** ^**2**^ **(df)**	***P*** **value**	**Mean (SD)**
Social and Legal Sciences and Humanities	6.0	131.73	0.287 (3)	0.963	5.77 (1.36)
Sciences	6.0	136.85			5.69 (1.75)
Health Sciences	6.0	131.73			5.72 (1.61)
Engineering and Architecture	6.5	133.54			5.50 (1.98)
Total					5.68 (1.71)
**2. To what extent are you collaborating in your research activity with some Spanish institution?**	**Median**	**Average rank**	***χ*** ^**2**^ **(df)**	***P*** **value**	**Mean (SD)**
Social and Legal Sciences and Humanities	4.0	182.81	6.878 (3)	0.076	4.08 (2.17)
Sciences	2.0	135.95			2.76 (1.88)
Health Sciences	2.0	130.35			2.68 (1.98)
Engineering and Architecture	2.0	121.09			2.41 (1.84)
Total					2.75 (1.94)
**3. To what extent are you collaborating in your research activity with your Spanish institution of origin?**	**Median**	**Average rank**	***χ*** ^**2**^ **(df)**	***P*** **value**	**Mean (SD)**
Social and Legal Sciences and Humanities	4.0	180.54	10.606 (3)	0.014*	3.77 (2.27)
Sciences	2.0	140.97			2.51 (1.89)
Health Sciences	1.0	125.78			2.13 (1.70)
Engineering and Architecture	1.0	114.13			1.91 (1.67)
Total					2.37 (1.86)
**b)** ***Efforts made by the host country to achieve collaboration***					
**1. To what degree does the country where you are carrying out your work help you to collaborate with other international institutions?**	**Median**	**Average rank**	***χ*** ^**2**^ **(df)**	***P*** **value**	**Mean (SD)**
Social and Legal Sciences and Humanities	6.0	123.12	1.452 (3)	0.693	5.38 (2.06)
Sciences	6.0	137.58			5.93 (1.31)
Health Sciences	6.0	128.66			5.77 (1.37)
Engineering and Architecture	6.0	141.91			6.00 (1.27)
Total					5.86 (1.37)
**2. To what degree does the country where you are carrying out your work help you to collaborate with Spanish institutions?**	**Median**	**Average rank**	***χ*** ^**2**^ **(df)**	***P*** **value**	**Mean (SD)**
Social and Legal Sciences and Humanities	5.0	148.92	1.231 (3)	0.746	5.31 (1.65)
Sciences	5.0	137.61			5.08 (1.64)
Health Sciences	5.0	130.30			4.82 (1.95)
Engineering and Architecture	5.5	127.69			4.71 (2.09)
Total					4.96 (1.80)
**3. To what degree does the country where you are carrying out your work help you to collaborate with your Spanish scientific institution of origin?**	**Median**	**Average rank**	***χ*** ^**2**^ **(df)**	***P*** **value**	**Mean (SD)**
Social and Legal Sciences and Humanities	6.0	165.92	2.817 (3)	0.421	5.46 (1.80)
Sciences	5.0	136.04			4.71 (1.91)
Health Sciences	4.0	130.15			4.47 (2.19)
Engineering and Architecture	5.0	127.71			4.35 (2.36)
Total					4.62 (2.06)
**c)** ***Efforts made by national scientific institutions to promote collaboration***					
**To what degree do the scientific institutions in Spain promote collaboration?**	**Median**	**Average rank**	***χ*** ^**2**^ **(df)**	***P*** **value**	**Mean (SD)**
Social and Legal Sciences and Humanities	2.0	130.54	1.436 (3)	0.697	2.54 (1.85)
Sciences	2.0	139.98			2.65 (1.60)
Health Sciences	2.0	128.34			2.45 (1.68)
Engineering and Architecture	2.0	130.53			2.38 (1.41)
Total					2.54 (1.61)

*P <0.05.

With regard to the levels of collaboration, researchers in health sciences indicated little collaboration with national scientific institutions and, specially, with their Spanish scientific institutions of origin (2.68 and 2.13, respectively; Table 1a), ranking second to last, and after researchers in engineering and architecture, with low total averages of 2.75 and 2.37, respectively (Table 1a). These data contrast with those referring to the degree of collaboration with international scientific institutions, which was better in all areas, including health sciences (above the total average of 5.72; Table 1a).

According to Table 1a, the area of social and legal sciences and humanities was at the top for the three items that measured levels of collaboration. As can be noted, in any case did health science point the first place. The best punctuation obtained by this area was in terms of collaboration with an international institution, which scored at the second place.

### Efforts made by the host country to achieve collaboration

In relation to the efforts made by the host country to facilitate collaboration with international, national and country of origin scientific institutions, the figures obtained for the total averages (5.86, 4.96 and 4.62; Table 1b) were above the average value (3.5). However, those corresponding to health sciences researchers were slightly below these total averages (5.77, 4.82 and 4.47; Table 1b).

According to Table 1b, the area of social and legal sciences and humanities also led in the items of ‘help to achieve collaboration by the host country with Spanish scientific institutions’ (5.31) and that of ‘help to achieve collaboration by the host country with Spanish scientific institutions of origin’ (5.46). On the other hand, engineering and architecture led the remaining item: ‘help to achieve collaboration by the host country with other international institutions’ (6.00; Table 1b). Therefore, the area of health sciences did not lead in any item and occupied the third place three times in this variable: 5.77, 4.82 and 4.47 (Table 1b).

### Efforts made by national scientific institutions to promote collaboration

In relation to this aspect, the total average of collaboration promoted by Spanish institutions was low (2.54; Table 1c). Those data corresponding to researchers working in the area of health sciences was even below those in the other variables (2.45; Table 1c). Sciences was the leader area in this variable (2.65; Table 1c), while health sciences ranked third.

### Type of collaboration maintained with scientific institutions in Spain

In relation to the types of collaboration (Table 2), conference attendance was over 50% only for researchers involved in social and legal sciences and humanities. Likewise, cooperation in joint research projects in this area reached a similar level. In all areas, the levels of other collaborations were well below this figure and these low percentages mainly concerned cooperation with the aim of conducting research projects, making joint publications and increasing the levels of conference attendance. Health sciences researchers ranked third for these types of collaboration, with low percentages of 23.9%, 13.6% and 14.8%, respectively.

**Table 2:** **Types of collaboration held with Spanish scientific institutions**

**Types of collaboration**	**Social and Legal Sciences and Humanities**	**Sciences**	**Health Sciences**	**Engineering and Architecture**
Elaborating joint publications	38.5%	25%	13.6%	11.8%
Applying for patents	0%	1.5%	1.1%	0%
Implementing joint research projects	46.2%	22.7%	23.9%	11.8%
Attending conferences	53.8%	22%	14.8%	8.8%
Obtaining research contracts	15.4%	6.1%	0%	8.8%
Execution/tutoring of PhD theses	15.4%	11.5%	5.7%	5.9%
Consultancy jobs	7.7%	3%	3.4%	0%
Informal contacts/business placements	7.7%	3.8%	3.4%	5.9%
Participation in networks by electronic means	7.7%	9.9%	3.4%	2.9%
Spin-off creation	7.7%	0%	1.1%	2.9%
Employee training	7.7%	5.3%	2.3%	0%
Funding procurement for the Spanish institutions	14.3%	1.5%	8.0%	5.9%
Creation of new or improved products or processes	0%	0.8%	1.1%	0%
Influence in socio-political changes	0%	0%	1.1%	0%
Recruiting of researchers for scientific institutions in Spain	15.4%	2.3%	0%	2.9%

### Profile of Spanish researchers abroad

**Figure 1:**
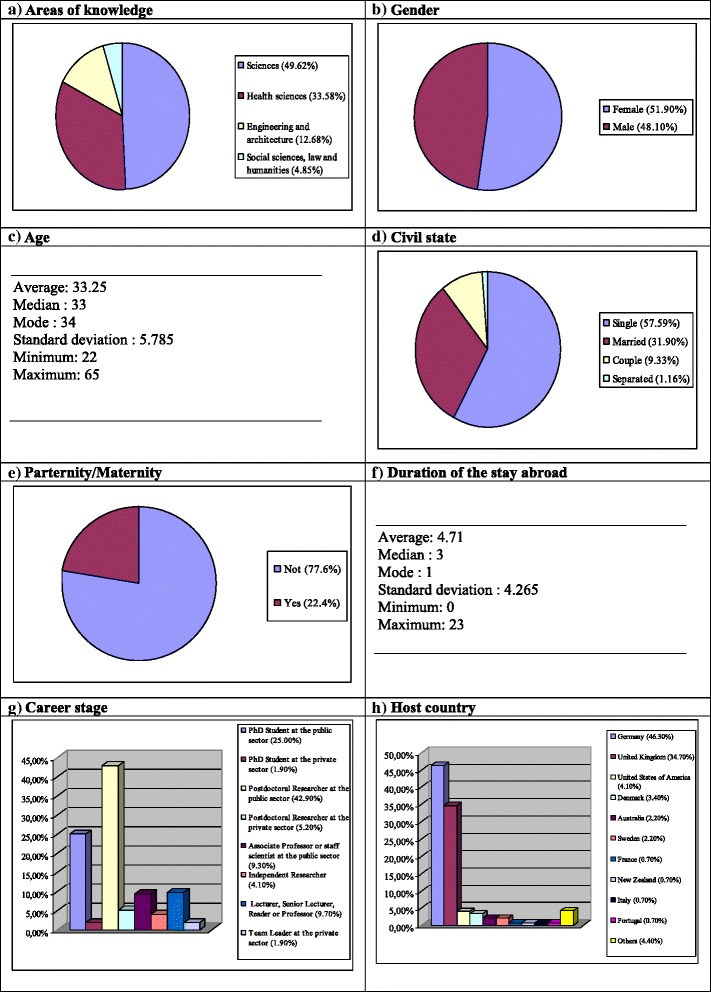
**Profile of Spanish researchers abroad.**

The profile of participants in the study is shown in Figure 1a-h and Tables 3 and 4. Briefly, the most represented areas of knowledge were sciences (133, 49.62%) and health sciences (90, 33.58%; Fig. 1a). Further, participants were almost equally distributed by sex (Fig. 1b), had a median age of 33 years (Fig. 1c), with the majority being single individuals and with no children (154, 57.59% and 208, 77.6%, respectively, Fig. 1d, e), and a median of 3 years spent abroad (Fig. 1f).

**Table 3:** **Reason for migration (distribution according percentage positive answers)**

**Reason**	**Number of researchers**	**Percentage**
Continuation/career advancement	196	73.10%
Lack of employment opportunities	136	50.70%
Professional recognition	104	38.80%
Lack of economic funding	97	36.20%
Postgraduate Studies	94	35.10%
Salary not commensurate with training and expectations	90	33.60%
Discover other cultures	65	24.30%
Inadequate level of social responsibility in the organisation	42	15.70%
Personal reasons	30	11.20%

**Table 4:** **Intention and possibility of returning to continue with scientific career in Spain (next 5 years)**

**Intention and possibility of returning**	**Number of researchers**	**Percentage**
I can and want to return	28	10.54%
I wish to return but I cannot	139	52.26%
I do not wish to return, although I can	33	12.40%
I do not wish to return and I cannot	66	24.80%
Total	266	100%

When asked about their career stage, all stages were represented in both the public and private sector, with a predominance of postdoctoral researchers (115, 42.9%) and PhD students (67, 25.0%) in the public sector (Figure 1g). Likewise, most participants were located in Germany (129, 46.3%), the United Kingdom (93, 34.7%), the United States (11, 4.1%), and Sweden (6, 2.2%; Figure 1h). As can be observed in Table 3, the main reason for the departure was the continuation and progress in research careers (196, 73.10%). Finally, over 60% (161) of the surveyed Spanish scientists abroad were willing to return to Spain (Table 4).

## Discussion

Our results show that, for researchers working in health, the figures in all areas assessed in the study were always below the total average, if collaboration with an international institution was excluded. In this regard, there was also a high level of cooperation between researchers settled abroad and international scientific institutions, which contrast with the limited collaboration held with national scientific institutions, including those of origin for each researcher.

This wide difference showed that the international ties made by health science researchers abroad are quite likely to exceed those resulting from the national network. As a consequence, their stay abroad might evolve from transitory to permanent, considering all the factors previously explained [11]. Besides affecting the probability of return, another important area, such as scientific productivity in Spain, can also be harmed, as mentioned previously [12].

In relation to the level of collaboration with the Spanish scientific institution of origin, the level of collaboration was on an even smaller scale than that considered for the national scientific institutions. This result does not improve the chance of returning either, if we consider that the scientists who had returned had maintained links with the organisation they were based at prior to their move abroad, as previously shown by Fontes [15].

The limited collaboration was also evident in the reduced percentage of health science researchers that have collaborated with national institutions and the nature of their collaboration, mainly concerning research projects, attending conferences and publications. Despite its limited number, this kind of collaboration in health sciences is coherent with the information obtained by Fontes [15], namely based on personal contacts, research projects and publications.

These main collaborations of researchers in the health sciences favours the good use of this scientist's knowledge and the attainement of positive evaluations in order to return to public institutions appointment positions. These factors may also be favoured by the execution/tutoring of PhD theses, although the share of this aspect is much smaller than in abovementioned collaborations. Regarding the types of collaboration considered professional experience for access to public institutions, companies or other private organizations, only the aspect related to funding procurement for Spanish institutions is somewhat meaningful.

It is, therefore, necessary to concentrate our efforts in improving main collaborations in order to encourage cooperation in other important areas such as spin-off creation, the provision of funding for national scientific institutions and the creation of new or improved products [23]. In addition, given the important role of research training programmes in creating and maintaining research networks [37], more efforts may also be concentrated in these programs as an added solution to strengthening previous collaborations.

Baruffaldi and Landoni [12] established that the probability of return increases with the decrease in the period of stay abroad, a more temporary professional situation and the reason for the departure not being related to better job opportunities. According to the figures obtained in this study, the Spanish researchers stayed abroad over 3 years on average, in agreement with previous studies [10]. Further, the majority of them worked in a temporary position (75% hold a postdoctoral position or lower) and the main reason for departure was the continuation and progress of their research careers.

According to these figures, we would include their professional situation as a positive indication of increased probabilities of return, whereas negative data includes an extended period of stay and the possibility of better job opportunities abroad. Despite this disparity in figures, over 60% of surveyed Spanish scientists abroad were willing to return, as discussed above. In addition, personal reasons would not be an obstacle for them to return (according to the data in our study, most of them were single and with no children). Therefore, SECTI agents and, in the case of researchers in health sciences, Sistema Nacional de Salud agents can promote actions to achieve this return through the fostering of collaboration. Within this new collaborative framework, it is quite possible that new professional opportunities may arise from the contribution of these scientists endowed with a greater international experience and knowledge.

Similarly, host countries are much more receptive to the collaboration with international and Spanish scientific institutions than the national scientific institutions when it comes to promoting collaboration with health science researchers in Spain. In this last sense, keeping a list of contacts among colleagues is also a valid means of getting scientists to return in the way Fontes [15] indicated. Consequently, the authorities and managers of the Sistema Nacional de Salud are advised to encourage these types of contacts. Of note, Fontes [15] also revealed the importance of establishing contacts with international associations of Portuguese scientists. In the case of Spain, this approach can be performed by employees of the Sistema Nacional de Salud through the recently established Spanish learned associations of the scientific diaspora: SRUK/CERU, CERFA, ACES and ECUSA.

We also show that the models that should be taken as examples in areas of knowledge related to the collaboration of Spanish scientists abroad did not show a much higher level than those concerning health sciences. The primary positions largely focus on the area of social and legal sciences and humanities, and therefore it would be interesting to assess whether this area is developing good practices in this sense.

The information obtained herein has implications for health management. An improvement in the collaboration with Spanish scientists abroad can bring about significant benefits to the Sistema Nacional de Salud. This collaboration could lead to an increase in human resources for this area through the re-establishment of contacts that could increase the possibility of working together, even if it would primarily be at a distance. Similarly, the quality of national human resources would also benefit from the enrichment of collaborating with those who have been further educated and who have gained experience abroad. In addition, integration into the labour market could be improved thanks to the awareness of the value added by these scientists in other countries, unseen by the thus far limited collaboration that has been found in this study. The awareness of their value can not only drive the discovery of new attractive opportunities for investment, but also lead to the creation of jobs, greater national and international scientific output, and a larger sharing of scientific knowledge. Likewise, collaboration between these scientists based abroad and the doctors willing to conduct research, but who have little time, can support the work of the latter by allowing them to also carry out experiments, and motivating them in all their research tasks. Finally, it would also be interesting to study the existing examples of international collaboration of national researchers working in Spain with scientists from other countries. The strengthening of this collaboration can be an important measure to enrich the investigations of national researchers without leaving their professional positions in Spain.

### Limitations

Although the data used to support these conclusions are reliable and have been obtained showing a clearer indication than that achieved by Fontes [15], our study has the limitation of having been conducted on a small number of Spanish researchers in the health sciences. Future studies will extend this number to ratify the validity of these results.

Most of the participants in this study have postdoctoral or lower job positions, with less freedom to establish the desired collaborations than other top positions. In addition, in this study, it was necessary to collect data from Spanish scientists by associations abroad due to the lack of knowledge of the total population of these Spanish researchers abroad. Therefore, the obtained results do not generalize to the entire collective. Similarly, this study shows the collaboration in a given moment of time, but not its evolution. For this reason, it is our intention to extend it with a longitudinal design in future works.

## Conclusions

Based on our results, we conclude that the national collaboration with Spanish health science researchers abroad is weak, which means that good practices to both benefit from the scientific knowledge potential and improve the likelihood of health science researchers returning from abroad are not put into practice. To achieve this, it is recommended to capitalise on the willingness of the host countries to collaborate at the international level and to further improve the promotion of collaboration through Sistema Nacional de Salud agents.

## Additional file

Additional file 1:
**Profile of Spanish researchers abroad.** (XLS 34 kb)

## Abbreviations

ACES: Asociación de Científicos Españoles en Suecia
CERFA: Comunidad de Científicos Españoles en la República Federal de Alemania
ECUSA: Comunidad de Españoles Científicos en Estados Unidos
R&D: Research and development
SECTI: Sistema Español de Ciencia, Tecnología e Innovación
SRKU/CERU: Society of Spanish Researchers in the United Kingdom/Comunidad de Científicos Españoles en el Reino Unido
